# The Molecular Basis of Different Approaches for the Study of Cancer Stem Cells and the Advantages and Disadvantages of a Three-Dimensional Culture

**DOI:** 10.3390/molecules26092615

**Published:** 2021-04-29

**Authors:** Danila Cianciosi, Johura Ansary, Tamara Y. Forbes-Hernandez, Lucia Regolo, Denise Quinzi, Santos Gracia Villar, Eduardo Garcia Villena, Kilian Tutusaus Pifarre, José M. Alvarez-Suarez, Maurizio Battino, Francesca Giampieri

**Affiliations:** 1 Department of Clinical Sciences, Polytechnic University of Marche, 60131 Ancona, Italy; d.cianciosi@pm.univpm.it (D.C.); nubansary@gmail.com (J.A.); luciaregolo@gmail.com (L.R.); denise81quinzi@gmail.com (D.Q.); 2 Nutrition and Food Science Group, Department of Analytical and Food Chemistry, CITACA, CACTI, University of Vigo, 36310 Vigo, Spain; tforbes@uvigo.es; 3 Research Center for Foods, Nutritional Biochemistry and Health, Universidad Europea del Atlántico, Isabel Torres 21, 39011 Santander, Spain; santos.gracia@uneatlantico.es (S.G.V.); eduardo.garcia@uneatlantico.es (E.G.V.); kilian.tutusaus@uneatlantico.es (K.T.P.); 4 Research Center for Foods, Nutritional Biochemistry and Health, Universidad Internacional Iberoamericana, Campeche 24560, Mexico; 5 Departamento de Ingeniería en Alimentos, Colegio de Ciencias e Ingenierías, Universidad San Francisco de Quito, Quito 170157, Ecuador; 6 King Fahd Medical Research Center, King Abdulaziz University, Jeddah 21589, Saudi Arabia; 7 International Research Center for Food Nutrition and Safety, Jiangsu University, Zhenjiang 212013, China; 8 Department of Biochemistry, Faculty of Sciences, King Abdulaziz University, Jeddah 21589, Saudi Arabia

**Keywords:** cancer stem cells, identification, isolation, enrichment, methodology, three-dimensional culture, surface molecular markers, functional assays

## Abstract

Cancer stem cells (CSCs) are a rare tumor subpopulation with high differentiation, proliferative and tumorigenic potential compared to the remaining tumor population. CSCs were first discovered by Bonnet and Dick in 1997 in acute myeloid leukemia. The identification and isolation of these cells in this pioneering study were carried out through the flow cytometry, exploiting the presence of specific cell surface molecular markers (CD34^+^/CD38^−^). In the following years, different strategies and projects have been developed for the study of CSCs, which are basically divided into surface markers assays and functional assays; some of these techniques also allow working with a cellular model that better mimics the tumor architecture. The purpose of this mini review is to summarize and briefly describe all the current methods used for the identification, isolation and enrichment of CSCs, describing, where possible, the molecular basis, the advantages and disadvantages of each technique with a particular focus on those that offer a three-dimensional culture.

## 1. Introduction

The presence of a rare cell population within the tumor mass, the CSCs, is one of the main causes of resistance to the common chemotherapeutic agents and radiotherapy, as well as of the metastatic process and of the presence of relapses; moreover, these cells would seem to be the real ones responsible for the tumorigenesis process [1]. The discovery of these high tumorigenic cells, made in 1997 by Bonnet and Dick in acute myeloid leukemia, placed the foundations for the development of a new model of tumorigenesis from a stochastic to hierarchical type [2]. Indeed, the hierarchical model that supports the theory that only CSCs are able to “initiate the tumor” is capable of justifying the widely proven heterogeneity of the tumor mass [3]. Since CSCs are considered to be among the main causes of resistance to chemo- and radiotherapy [1], it seems obvious that finding therapies aimed at targeting this rare population could help to decrease this phenomenon and consequently avoid metastasis and relapses over time. In order to develop or investigate the effect of natural or synthetic molecules/compounds able to target these CSCs, the first step has been their identification and isolation. Over the years (since their discovery), different methodologies have been used and developed for the identification, isolation and enrichment of these cells. In this mini review, the different methodologies will therefore be briefly discussed, which are basically divided into surface marker assays and functional assays, paying greater attention to the molecular aspects (especially for surface marker assays) and to the sphere forming assay methodology, which moreover allows the development of a three-dimensional culture model that better mimics what really happens in vivo, highlighting the advantages and disadvantages of this culture method compared to a traditional two-dimensional culture.

## 2. Methods of Identification, Isolation and Enrichment of CSCs

Different methods are used for the study of CSCs from both primary tumors and cell lines, some of which are based on the expression patterns on the cell surface, while others instead depend mainly on some peculiar functionalities and characteristics of CSCs. The main methods are summarized in Table 1 and they will be further discussed in the following sections.

### 2.1. Surface Markers Screening Assays

Several surface proteins have been identified over the years in the cell membrane of CSCs, also referring to the fact that the first study on CSCs by Bonnet and Dick (1997) was based on the identification of the Cluster of differentiation (CD) 34^+^/CD38^−^ phenotype [2]; among the different surface markers used by researchers in the last years to identify, isolate and enrich CSCs in other tumor types, the most common are CD133, CD24, CD44, CD13, CD166 and Epithelial cell adhesion molecule (EpCAM), which can be used alone or in combination [4] (Table 2).

The identification and the subsequent isolation and enrichment of CSCs through methods based on the presence of these surface markers, on the one hand, offers different advantages, including the simplicity of execution of these methods, but on the other hand it has important disadvantages. Among these is the fact that very often these surface markers are not strictly specific to CSCs. In fact, very frequently, many markers among those mentioned are also found in embryonic stem cells (ESCs) and adult stem cells (ASCs) as well as in normal tissues or cells [5]. For this reason, the presence of surface markers is frequently associated with some intrinsic properties of CSCs, such as the ability to grow detached from niche elements [6] or a greater specific enzymatic activity—for example, ALDHs [7].

CD133, also known as prominin-1 or AC133, is the most studied surface antigen in CSCs and the most used for their study. It is a 97 kDa membrane glycoprotein, formed by five transmembrane portions connected by two intracellular and two extracellular loops [8]. In humans, CD133 gene is located on chromosome 4 [9], its biological function, both at the physiological and pathological levels, is not fully understood; its function has been hypothesized in the organizing process of membrane formation and seems to be involved in various signaling pathways, including those related to stem properties (Wingless-related integration site (Wnt)/β-catenin) [10] and cell cycle regulation (phosphatidylinositol 3-kinase (PI3K)/AKT/mammalian target of the rapamycin (mTOR)) [11]. Among the different types of cancer in which this membrane glycoprotein acts as a marker are pancreas, colon, liver, prostate lung and brain [8]. CD133 is expressed both in ESCs [12] and in ASCs, in particular in the hematopoietic [13], neural [14] and prostatic ones [15]. The expression of CD133 on the other hand in nonstem cells and therefore in normal tissues is rather rare [5]. CD24, also called heat stable antigen (HSA), is a small surface sialoglycoprotein, whose physiological role seems to be linked to the cell–extracellular matrix (ECM) and cell–cell interaction functions, although different glycosylations of this membrane protein are attributed to different cell types and different functionalities, with some still to be clarified [16]. In humans, the CD24 gene is located on chromosome 6 [17]. CD24 is highly expressed in kidney, bladder, breast and ovary and is often used in association with CD44 for the study of CSCs [18]. This marker has also been found in ESCs [19] and intestinal ASCs [20]; expression in normal cells or tissues is quite rare and has been found in lymphocytes [21] and neural cells [22].CD44, also called P-glycoprotein 1, in humans is encoded by a gene on chromosome 11. It is an 85–200 kDa protein, with different weights for different isoforms [23]. It was initially identified as a membrane receptor of hyaluronic acid and was subsequently observed to also act as a receptor for other ligands, such as collagen, osteopontin and matrix metalloproteinases (MMPs). For this reason (receptor of MMPs), it would seem to play a crucial role in the metastatic and angiogenetic potential of cancer cells and in particular of CSCs [24], for which it represents a marker, especially in colon, prostate, stomach, ovary and breast tissues [25]. CD44 has not been found to be expressed in ESCs [5], but its expression has been observed, for example, in hematopoietic [26], adipose [27] and mesenchymal ASCs [28]. It has been observed that some cells derived from lymphatic [29] and epithelial tissues also express some isoforms of CD44 [30]. CD13, also called alanyl aminopeptidase, in humans is encoded by a gene on chromosome 15 [31]. It is a membrane protein with different functions—from the presentation of the antigen, to the enzymatic cutting of peptides, to the involvement in various pathways that mediate proliferation, motility and cell adhesion; this protein also seems to be involved in the processes of angiogenesis and epithelial–mesenchymal transition (EMT) [32]. It represents a marker of identification and isolation of CSCs especially in liver cancer [33]. CD13 was not found to be expressed in ESCs [5], while it is expressed in mesenchymal ASCs [34], while again its expression in normal cells or tissues is rare [5]. CD166, also known as activated leukocyte cell adhesion molecule (ALCAM), is a transmembrane glycoprotein of 100–105 kDa, that in humans is encoded by a gene on chromosome 3 [35]. The most important function of this surface protein is to mediate adhesion interaction [36]. It represents a surface marker particularly for CSCs deriving from colon, lung, head and neck cancers [37]. CD166 in ESCs has a weak expression [5], while in ASCs it has been found to be expressed mainly in intestinal [38] and adipose ones [39] and also in many normal epithelial cells [40]. EpCAM, whose gene is found on chromosome 2, is a transmembrane glycoprotein involved in migration, cell signaling, Ca^2+^-independent cell adhesion and proliferation. For these reasons, it seems to be involved in carcinogenesis and metastasis processes [41] and it is considered to be a new marker for CSCs, especially in breast, colon and liver cancers [42]. EpCAM is also expressed in ESCs and some intestinal-derived ASCs [43], as well as in some tissues and nonstem epithelial cells [5].

**Figure 1:**
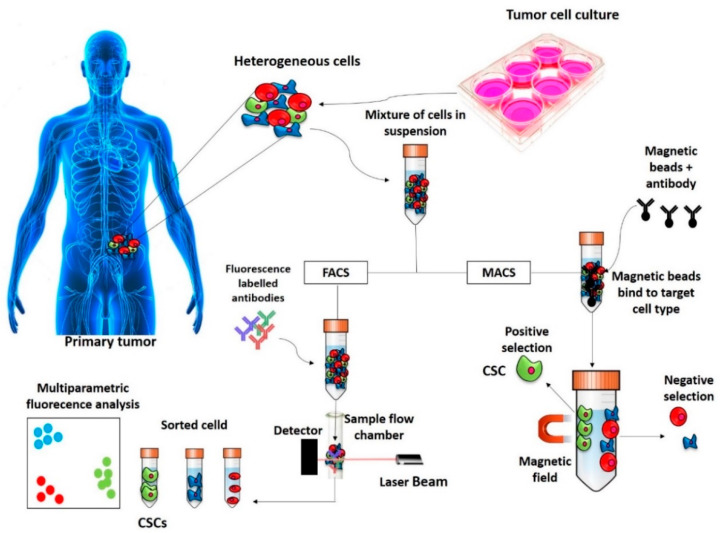
Representation of isolation of CSCs based on surface markers. The CSCs are isolated based on CSC marker expressions by FACS and MACS techniques. CSC—cancer stem cell; FACS—fluorescence-activated cell sorting; MACS—magnetic-activated cell sorting.

Basically, there are two methods used for the isolation of CSCs based on the detection of these and/or others surface markers (Figure 1): Fluorescence-activated cell sorting (FACS) and Magnetic-activated cell sorting (MACS). 

#### 2.1.1. FACS

FACS involves the use of fluorescently labelled antibodies targeting the specific surface markers of the CSCs and subsequent separation of the fluorescent vs. nonfluorescent population [44]. In general, FACS is a technique that allows measuring the chemical and physical characteristics of cells or, by extension, of other biological particles. It allows the measurement of a series of parameters, such as cell size [45], granularity [46], content of pigments [47], DNA, RNA [48], membrane potential [49], surface and intracellular receptors [50], enzymatic activities [51] or protein phosphorylation [52]. The study of CSCs with FACS is based on surface markers: the cells are discriminated on the basis of the presence or absence of certain surface markers targeted with fluorescent antibody ligands. One of the advantages of this method is that it is a multiparametric analysis and therefore allows the use of different antibodies at the same time [44]. One of the major advantages of this technique is that a large number of cells is needed [4].

#### 2.1.2. MACS

MACS is a cell separation technique based on the use of monoclonal antibodies conjugated with magnetic beads. After incubating the magnetic beads with the cell suspension, the cells presenting the selected surface marker are then attracted and separated by a magnet, which allows them to bind to a column, from which they are then eluted. One of the major advantages of this technique is that it is an easy method that allows the use of few cells, while one of the major disadvantages of this technique is that there is the possibility of selecting the cells in a single-parameter manner [53].

### 2.2. Functional Screening Assays

**Figure 2:**
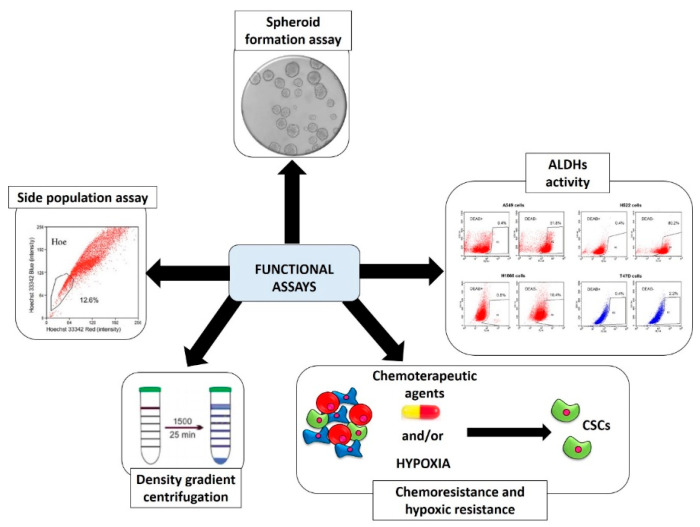
The most used functional assays for the identification, isolation and enrichment of cancer stem cells. CSCs: cancer stem cells. The most used functional assays for identification, isolation and enrichment of CSCs are spheroid formation assay, ALDHs activity, selection for chemo and/or hypoxic resistance, density gradient centrifugation and side population assay.

As previously mentioned, CSCs have some functional properties that distinguish them from the remaining tumor cells; these properties have been exploited by researchers to develop methods to identify them, isolate them from the tumor-bulk and allow their enrichment. The most used are schematized in Figure 2 and briefly described in the next paragraphs.

#### 2.2.1. Spheroid Formation Assay

Spheroid formation assay, as will be discussed in detail in Section 3, offers the advantage of working with a three-dimensional (3D) culture and better mimicking the properties of a tumor tissue [54]. This technique is founded on the cultivation of a single-cell suspension in a serum-free medium supplemented with certain growth factors (epidermal growth factor (EGF) and human basic fibroblast growth factor (b-FGF)) in low-attachment culture systems [55]. This concept is based on the anchorage-independent growth properties of CSCs cells that are able to survive after being detached from niche elements and form successive spheroids through the clonal proliferation and not for simple aggregation [56]. This does not occur in the population of nonstem cell tumor cells, which undergo a cell death phenomenon called anoikis under the same culture conditions [57]. Other similar cultivation systems allow the enrichment of these cells on different kind of scaffolds such as agar, agarose, matrigel, polycaprolactone fibrous and could have further advantages as they offer the opportunity, for example, to carry out cocultures or to study the interaction with the ECM. One of the major disadvantages of this technique is that there is a moderate cellular heterogeneity with the presence in the culture also of differentiated cells [58].

#### 2.2.2. Aldehyde Dehydrogenases (ALDHs) Activity

It has been observed that CSCs have a greater activity of these enzymes, and therefore they have been considered as a functional parameter for their isolation that is carried out either through FACS analysis [59] or through the Aldefluor assay, which through a chemical reaction converts the ALDH substrates into a fluorescent product—the BODIPY-amino acetate [60]. ALDHs are enzymes mainly involved in the detoxification of both endogenous and exogenous aldehydes. In humans, 19 members of the ALDH family have been identified with multiple functions: structural, antioxidant, regulatory and catalytic ones. In cancer and in particular in CSCs, which, as previously mentioned, are more highly expressed (in particular the ALDH1A1 and ALDH1A3 isoforms), they contribute to the phenomenon of chemoresistance and are also used as prognostic indicators. One of the disadvantages of this method for the study of CSCs is that many nontumor stem cells have also been found to have a high activity of ALDHs [61].

#### 2.2.3. Side Population (SP) Assay

This method is based on the assumption of the presence on the surface of the CSCs of ATP Binding Cassette (ABC) transporters, which are able to extrude the fluorescent DNA-binding dye Hoechst 33342 from the cell. Cells are separated on the basis of this principle by the FACS into SP^+^ (ability to extrude Hoechst 33342) and SP^−^ [62]. The presence of ABC transporters on the surface of the CSCs is strictly associated with a higher chemoresistance; specifically, ABCG1, ABCC1 and ABCG2 act as efflux pumps and extrude the chemotherapeutic drugs from inside to outside of the cells [63]. One of the disadvantages of this technique for the identification/isolation/enrichment of CSCs is the lack of real standardization of the method [4].

#### 2.2.4. Chemoresistance and Hypoxic Resistance

The approach of this type of method is based on the exposure of the population to chemotherapeutic agents [64] and/or to hypoxic condition [65], allowing the survival of only those cells that resist on these conditions. The advantage is the simplicity of the method, while the disadvantages of this method are that it does not offer total homogeneity of the selected cells and that there is not a standardized protocol [4].

#### 2.2.5. Physical CSCs Properties (Density Gradient Centrifugation)

This separation method is based on the physical properties of CSCs and uses different density gradients (Ficoll, Percoll abd Histopaque) that result in different degrees of efficacy of isolation/enrichment [66]; the advantage of this is the ease and speed of the experiment, while one of the disadvantages is the low homogeneity [4].

#### 2.2.6. Other Functional Isolation Methods

Among other lesser used functional isolation methods, there are the label-retaining methods (lipophilic dyes) [67], the CSC selection by natural killer (NK) cells [68,69], the promoter-driven fluorescent protein expression [70], the evaluation of the intracellular concentration of reactive oxygen species (ROS) [71] and of the mitochondrial membrane potential (Δψm) [72] and the autofluorescence [73].

The label-retaining methods are based on the ability of CSCs to divide asymmetrically, resulting in slower dividing cells with respect to the rest of the tumor population. For this reason, if the cells are labeled with specific lipophilic and fluorescent dyes that bind to cell membranes, CSCs will be fluorescent for a longer time than differentiated cells [67]. This method is basically used to identify/isolate/enrich CSCs from osteosarcoma [74] and breast cancer [75].

CSC selection by NK cells, a subtype of cytotoxic lymphocytes, is based on the recent observation that CSCs are more sensitive to be lysed by NK cells [68,69]. This selective methodology has been proven in oral squamous carcinoma and breast cancer, but there are still no clinical applications of this technique [76].

The promoter-driven fluorescent protein expression assay is a slightly more complicated technique as it allows the study of CSCs through the bond of fluorescent labeling (GFP) with a promoter of some genes typical of stem cell expression patterns, such as Octamer-binding proteins (Oct)-4, Sox2 and Nanog, which are more specific than the classic surface markers [70]. This method has limitations in that the techniques are quite complicated and there are no standardized protocols. Sometimes, this method is also used with other genes that are specific to some types of CSCs, such as, for example, for Lgr5, which is specific, for example, for colon, breast [77] and liver [78]. Furthermore, it has been observed that it is possible to develop organoids from single cells expressing this marker and this could represent a further method for the study of CSCs [79].

Regarding the selection through the assessment of the intracellular accumulation of ROS, in recent times it has been observed that, within the tumor population, CSCs have lower levels of ROS and higher levels of antioxidant enzymes and this is the basis of an isolation method through a fluorescent staining system (FACS) that identifies ROS^+^ cells and is able to separate them from the ROS^−^ population [71].

Δψm has been proven to be a marker of cellular tumorigenicity, related to greater resistance to apoptosis and greater angiogenic potential. It was recently observed that in the heterogeneous tumor population, the mitochondrial membrane potential was even higher in cells with stem-like surface markers. Additionally, in this case, through fluorescence methods (FACS) it is possible to identify, enrich and isolate these CSCs that have a higher mitochondrial membrane potential [72].

The technique based on autofluorescence is very recent and represents a new approach never used in the clinical area and which still needs further confirmation. In recent studies, it has been observed that some CSCs (particularly from gliomas) possess intrinsic fluorescence (excitation at 488nm with consequent emission at 520nm). These autofluorescent cells would possess self-renewal and higher tumorigenic capacity in vivo and upregulate typical stem genes (Nanog, Notch1, Oct4 and Sox2), characteristics attributable to the tumor stem cell phenotype [73].

## 3. Advantages and Disadvantages of a Three-Dimensional Culture in the Study of CSCs

Among all the methods described, in the study of a tumor cell model, the one that offers the greatest advantages from the point of view of proximity to reality is the spheroid formation assay, which offers the advantage of working with a cell model different from the common monolayer. It should be noted that this method is not the only one used in cancer research that allows the use of a 3D model, but very few others allow the study of CSCs. Among these, there is the hanging drop method (very complex and little used) and models that involve the use of scaffolds (also mentioned in Section 2.2.1.) [80].

It is currently estimated that over the 80% of preclinical in vitro studies are still assigned to two-dimensional (2D) culture techniques that offer quickness, convenience and extensive literature on protocols and for data comparison. Very often, it happens that when the results obtained from these studies are translated to in vivo conditions or even to clinical trials, the same treatments that had shown efficacy in monolayer culture models have no or very weak efficacy in animals or patients. This phenomenon has in part been attributed over the years to the fact that in cells in 2D culture some fundamental aspects lack, such as tumor architecture, cell–cell interactions, thus failing to reflect the real pathophysiology of cancer cells. The first reports of 3D cultures were in the late 1980s [81]. One of the methods of propagation and therefore enrichment of CSCs, as seen in the previous paragraph, is the in vitro sphere forming assay or three-dimensional culture through the use of scaffolds composed of different materials or the hanging drop method. These enrichment methods, therefore, offer, in addition to the opportunity to work with CSCs (strictly involved in the processes of tumorigenesis, chemo- and radioresistance and the presence of metastases), the opportunity to work with a three-dimensional model that better mimics what actually happens in vivo, certainly better than a normal monolayer culture.

**Figure 3:**
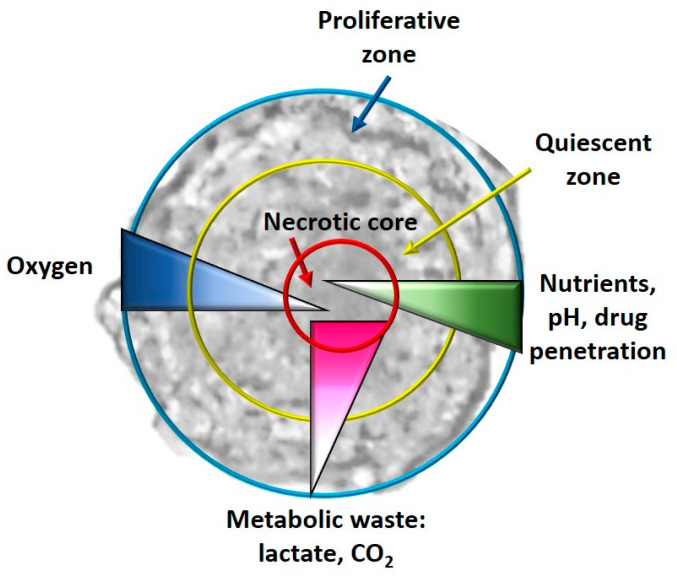
Graphical representation of the different zones (proliferative, quiescent and necrotic) and the different gradients (oxygen, nutrients, drug penetration and metabolic waste) in a typical spheroid.

Among the main differences between 2D and 3D culture models are the cell morphology [82], cell-matrix [83] and cell–cell interactions [84], proliferation rate [85], and microenvironment due to the ECM that is formed [86], distribution of nutrients, oxygen and different metabolic waste [87], different signal transduction, different gene and protein expression [88], reduced sensitivity to chemotherapy in 3D cultures [89], hypoxia and consequent processes [90] (Figure 3).

Another advantage offered by 3D cell cultures could be the partial replacement of the in vivo models allowing the use of a smaller number of animals—for example, with multicellular spheroid systems (tumor-fibroblast-endothelial cells) that mimic the tumor heterogeneity and the vascularization phenomena [91].

Cells grown in monolayers have equal access to nutrients, growth factors and oxygen, which is not what happens in vivo; through 3D cellular models, it is possible to recreate this limitation. In addition, the monolayer cells attached to the surface in a unilateral way induce an unnatural polarity for many cells, and each cell of the monolayer culture also has the same metabolic status, which is rare in vivo (where there are in fact proliferating cells, quiescent and necrotic ones); it is possible to recreate this situation in a spheroid model, especially due to hypoxic situations [86].

In the following paragraphs, we will consider the main differences between 3D and 2D cellular models, with implications in the culture of CSCs:

- Hypoxia and metabolism in 3D culture models: In general, in the tumor masses a condition of hypoxia is present in vivo, especially for the cells located in the core of the tumor bulk; this condition is impossible to reproduce in a monolayer culture system. When the cells, especially tumor cells, are grown with 3D culture methods, the formed spheroids simulate the tumor mass. It has been observed that real oxygen gradients, nutrients and metabolites are generated in spheroids miming those observed in vivo, even if, for example, cellular heterogeneity is not created in single-cell spheroids, but it is, however, a system that is the closest to the real one. In fact, in the spheroids, as happens in vivo, it has been observed that in their cores, there are cells that live in conditions of hypoxia and have gene alterations compared to all the other tumor mass [92]. Furthermore, necrotic cells are present in the core of the spheroid, as happens in vivo, and quiescent cells are present immediately outside the necrotic core. As regards the hypoxia-CSCs relationship, this is a fundamental parameter as hypoxia is an essential condition for the formation and expansion of this cellular niche [93]. In some studies, it has been observed that these hypoxic conditions activate some pathways closely related to CSCs, such as Wnt, Hedgehog (Hh) and Notch [94]. Cells grown in 2D, on the other hand, have uniform contact with oxygen and therefore it is impossible to observe an oxygen gradient [86].
- Angiogenesis in 3D CSC models: Angiogenesis is the process which, in tumor bulk, supplies oxygen and nutrients to cancer cells, allowing their growth, invasion and metastasis. CSCs are closely linked to the phenomenon of angiogenesis [95]. From the point of view of the expression of some genes involved in angiogenesis, 3D multicellular models with endothelial cells are, in particular, much closer to the in vivo conditions than a normal 2D culture, where it is not possible to recreate the angiogenic process [96].
- EMT in 3D culture models: Another difference in the gene expression patterns between 2D and 3D cultures is found in the EMT process, in which CSCs play a fundamental role. In 2D models the shape of cells is flattened, while in 3D cultures the cells take on shapes more similar to what they really are, forming aggregates and cell–cell interactions that are fundamental characteristics for studying this process, interactions that are absolutely not recreated in a 2D cellular model [97].
- Chemoresistance in 3D culture models: CSCs play a fundamental role in chemoresistance, which is one of the main causes of therapeutic failures in cancer treatments. 3D culture models could better simulate the in vivo situation for studying drug penetration, response and resistance. Indeed, it has been observed that the 3D cultures themselves and even more those that allow the enrichment of CSCs possess greater resistance to drugs related to the type of architecture of the spheroid [98]. In 2D cultures, for example, the size of the surface (surface/volume ratio) is very large and this allows an easy absorption of the treatment, which is not the case of a 3D culture or even in vivo [99].
- Among the disadvantages of 3D cultures, there are the costs and times that are higher than 2D culture systems, poor reproducibility, standardization, automation and comparison with literature data. Furthermore, adapting the protocols used in 2D to the conditions of 3D is not easy and there is low standardization, especially for cytotoxicity tests, but also for other analyses, such as Western blot. Moreover, greater ability and expertise in sample handling are also required [100].

The main differences between 2D and 3D cultures are shown in Table 3.

## 4. Conclusions

In recent years, the discovery of CSCs, a cell population within the tumor mass that possesses greater tumorigenicity as well as high chemo- and radioresistance and the ability to develop metastases and relapses over time, has led to the development of different methodologies essentially based on two principles (the presence of specific surface markers and specific functional characteristics) for the study of CSCs. The development of these techniques therefore allows better evaluation of the effect of natural and synthetic compounds and the synergistic effect of different compounds on this rare tumor subpopulation as well as the development of new drugs that specifically target CSCs, which are resistant to the most common chemotherapeutic drugs and radiotherapy. In this review, the main methods used for the identification/isolation/enrichment of CSCs have been summarized and briefly described, mentioning their advantages and disadvantages. As it has been possible to observe, there is not an exclusive technique that presents only advantages, but all the methods discussed present disadvantages and difficulties. The study of CSCs, therefore, requires greater interest in improving the weaknesses of the methods, the major ones being the presence of a high heterogeneity of the selected cell population and the poor standardization of protocols for the implementation of these methods. Greater attention in recent years has been focused on those methods that allow, in addition to studying a population with chemoresistance characteristics, the use of a three-dimensional cellular model. This culture method, in fact, being closer to the in vivo tumor system, offers numerous advantages, while bringing some disadvantages, among which the greatest is the poor standardization of protocols. The development and improvement of the current techniques used for the identification/isolation/enrichment of CSCs, which leads to a standardization of protocols, to a more homogeneous selection of the tumor stem cell population is of fundamental importance in the development of new therapies that selectively target CSCs to decrease one of the major causes of therapeutic failure in cancer treatment, which is the chemoresistance.

## Figures and Tables

**Table 1:** Advantages and disadvantages of the different methods for the study of CSCs.

Methods	Advantages	Disadvantages
Surface markers with FACS ^1^	-multiparametric analysis	-large number of cells is needed
Surface markers with MACS ^2^	-easy method -use of few cells	-single-parametric analysis
Spheroid formation assay	-use of a 3D cellular model	-moderate cellular heterogeneity
ALDHs ^3^ activity	-consolidated method	-many nontumor stem cells have also high activity of ALDHs
SP assay	-high specificity	-lack of real standardization of the method
Chemoresistance and hypoxic resistance	-simplicity of the method	-lack of protocol standardization -moderate cellular heterogeneity
Physical CSCs properties	-simplicity and speed of the experiment	-low homogeneity

^1^ FACS: fluorescence-activated cell sorting; ^2^ MACS: magnetic-activated cell sorting; ^3^ ALDHs: aldehyde dehydrogenases.

**Table 2:** The most common surface markers of CSCs.

CSCs Marker	Expression in ESCs	Expression in ASCs	Expression in Normal Tissues/Cells	Expression in CSCs
CD133	yes	hematopoietic, neural, prostatic	rare	pancreas, colon, liver, prostate, lung, brain
CD24	yes	intestinal	rare, neural cells, lymphocytes	kidney, bladder, breast, ovary
CD44	no	hematopoietic, adipose, mesenchymal	yes, lymphatic and epithelial tissue	colon, prostate, stomach, ovary, breast
CD13	no	mesenchymal	rare	liver
CD166	weak	intestinal, adipose	epithelial cells	colon, lung, head and neck
EpCAM	yes	intestinal	rare	breast, colon, liver

**Table 3:** Main differences between 2D and 3D cultures.

Advantages and Disadvantages	2D Culture	3D Culture
Chemical gradient formation	−	+
Physiological architecture	−	+
3D cell migration/interaction	−	+
Drug resistance	−	+
In vivo-like gene expression	−/+	+
Protocol standardization	+	−
Reproducibility	+	+/−
Comparison in scientific literature	+	+/−

## Data Availability

Data sharing is not applicable to this article. No new data were created in this study.
